# Effects of colchicine use on ischemic and hemorrhagic stroke risk in diabetic patients with and without gout

**DOI:** 10.1038/s41598-022-13133-0

**Published:** 2022-06-02

**Authors:** Jun-Jun Yeh, I-Ling Kuo, Hei-Tung Yip, Min-Yuan Hsueh, Chung-Y. Hsu, Chia-Hung Kao

**Affiliations:** 1 Department of Family Medicine and Medical Research, Ditmanson Medical Foundation Chia-Yi Christian Hospital, Chiayi, Taiwan; 2 China Medical University, Taichung, Taiwan; 3 Department of Nutrition, Ditmanson Medical Foundation Chia-Yi Christian Hospital, Chiayi, Taiwan; 4 Management Office for Health Data, China Medical University Hospital, Taichung, Taiwan; 5 College of Medicine, China Medical University, Taichung, Taiwan; 6 Graduate Institute of Biomedical Sciences and School of Medicine, College of Medicine, China Medical University, No. 2, Yuh-Der Road, Taichung, 404 Taiwan; 7 Center of Augmented Intelligence in Healthcare, China Medical University Hospital, Taichung, Taiwan; 8 Department of Nuclear Medicine and PET Center, China Medical University Hospital, Taichung, Taiwan; 9 Department of Bioinformatics and Medical Engineering, Asia University, Taichung, Taiwan

**Keywords:** Cardiology, Endocrinology, Neurology, Rheumatology

## Abstract

This study aimed to determine the effect of colchicine use on the risk of stroke among patients with diabetes mellitus (DM). We retrospectively enrolled patients with DM between 2000 and 2013 from the Longitudinal Health Insurance Database and divided them into a colchicine cohort (n = 8761) and noncolchicine cohort (n = 8761) by using propensity score matching (PSM). The event of interest was a stroke, including ischemic stroke and hemorrhagic stroke. The incidence of stroke was analyzed using multivariate Cox proportional hazards models between the colchicine cohort and the comparison cohort after adjustment for several confounding factors. The subdistribution hazard model was also performed for examination of the competing risk. The colchicine cohort had a significantly lower incidence of stroke [adjusted hazard ratios (aHR), 95% confidence intervals (95%CI)] (aHR = 0.61, 95%CI = 0.55–0.67), ischemic stroke (aHR = 0.59, 95%CI = 0.53–0.66), and hemorrhagic stroke (aHR = 0.66, 95%CI = 0.53–0.82) compared with the noncolchicine cohort. Drug analysis indicated that patients in the colchicine cohort who received colchicine of cumulative daily defined dose (cDDD) > 14 and duration > 28 days had a lower risk of stroke and ischemic stroke compared with nonusers. The colchicine cohort (cDDD > 150, duration > 360 days) also had a lower risk of stroke, ischemic stroke, and hemorrhagic stroke. The cumulative incidence of stroke, ischemic stroke, and hemorrhagic stroke in the colchicine cohort was significantly lower than that in the noncolchicine cohort (log-rank *P* < 0.001). However, the subdistribution hazard model reveal the colchicine was not associated with the hemorrhagic stroke in DM patients without gout (aHR = 0.69, 95%CI = 0.47–1.00). Colchicine use with cDDD > 14 and duration > 28 days was associated with lower risk of stroke and ischemic stroke, and colchicine use with cDDD > 150 and duration > 360 days played an auxiliary role in the prevention of stroke, ischemic stroke, and hemorrhagic stroke in patients with DM. The colchicine for the hemorrhagic stroke in DM patients without gout seem to be null effect.

## Introduction

The stroke are closely associated with the system inflammation and these diseases having the high level of the C-reactive protein (CRP) and inflammatory cytokines such as interleukin-6 (IL-6)^1,2^. Meanwhile, previous data provide evidence that nucleotide-binding domain and leucine-rich repeat protein-3 (NLRP3) inflammasome mediated inflammation is implicated in the etiology of DM and promotes DM-induced endothelial inflammation and atherosclerosis^1,2^. Thus, the NLRP3 may contribute to early neurological deterioration in stroke patients with DM. The colchicine displays its anti-inflammatory effect through inhibition of interleukn-1 (IL-1) and IL-6, granulocyte-macrophage colony-stimulating factor (GM-CSF), and NLRP3 inflammasome, and it is used to prevent the complication of the coronary artery disease (e.g., cardiac arrhythmia). Moreover, the colchicine use are associated with the lower risk of the recurrent ischemic stroke among acute non-cardiogenic ischemic stroke patients^3^. Altogether, the colchicine may play a role for attenuating the risk of stroke in patients with DM^3,4^.

A random-effects meta-analysis model was applied by Masson et al.^5^. They analyzed nine eligible trials of colchicine therapy involving a total of 6630 patients, (colchicine cohort, n = 3359; noncolchicine cohort, n = 3271). The stroke incidence was lower in the colchicine cohort than in the noncolchicine cohort^5^. However, Khandkar et al. performed a systematic review and meta-analysis of studies and concluded that the results for the effect of colchicine on stroke incidence were inconclusive^6^. Therefore, the relationship between the colchicine and the stroke is to be debated.

In Taiwan, colchicine is a historic treatment for gout, Familial Mediterranean Fever and its associated complication, amyloidosis. In recent years gout patients who have been taking colchicine for years have demonstrated novel applications within oncology, immunology, cardiology and dermatology^7,8^. The increasing prevalence of gout is associated with several factors including increasing incidence of metabolic syndromes and, in turn, DM. Meanwhile, the rising pace of aging across the globe is rising joint-related disorders such as joint pain, and gouty arthritis is estimated to enhance the growth of colchicine. Moreover, the micro- and macrovascular complications of DM are predisposing factors of the stroke, leading to the high frequency of the stroke among the those patients with DM. Up to today, no English literature has focused on the association between colchicine use and stroke (including ischemic and hemorrhagic strokes) risk among DM cohort. Thus, we investigated this association in patients with DM from the general population.

## Materials and methods

### Data source

Our data source was the Longitudinal Health Insurance Database 2000 (LHID2000), which is a representative subset of Taiwanese National Health Insurance Research Database (NHIRD) and contains records of one million people randomly sampled from 23 million beneficiaries of the universal health insurance program in Taiwan. All diagnostic codes for the claims are recorded according to the *International Classification of Diseases, 9th Revision, Clinical Modification* (ICD-9-CM). Personal identification information was anonymized to protect the privacy of the insured subjects. Because the NHIRD data set comprises anonymized secondary data, informed consent was not required. This study was approved by the Research Ethics Committee of China Medical University and Hospital in Taiwan (CMUH104-REC2-115-AR-4).

### Study design and participants

We designed a population-based retrospective cohort study by using the LHID2000 dataset to examine the association between colchicine use and stroke in patients with DM. Patients over 18 years of age with DM (ICD-9-CM code 250) from 2000 to 2013 were included as the target population. The colchicine cohort comprised patients who were prescribed colchicine (Anatomical Therapeutic Chemical (ATC) code M04AC01) for at least 28 days following the DM diagnosis. The date of the first prescription of colchicine was defined as the index date. Patients in the noncolchicine cohort were randomly selected from DM patients who were never prescribed colchicine during the study period. The index date for the noncolchicine cohort was randomly assigned. Patients with stroke history before the index date were excluded from the study.

### The classification of stroke

Based on Trial of Org 10172 in Acute Stroke Treatment (TOAST) criteria, the ischemic stroke included the (1) large-artery atherosclerosis, (2) cardioembolism, (3) small-vessel occlusion, (4) stroke of other determined etiology, and (5) stroke of undetermined etiology. In the Goldstein et al. study, 73% of patients with code 434.11 had embolic strokes, and 47% of those with code 436 had an identified stroke cause. Of patients with code 434.91, 39% had stroke of uncertain cause, 25% "lacunar," 17% atherothrombosis, and 15% embolism^9,10^. Information on the etiology and subtypes of ischemic stroke was not available in the NHIRD. We cannot discriminate the subtypes of ischemic stroke according to the TOAST classification. However, in accordance with Goldstein et study, we designated the ICD-9CM in the appendix Table 4.

In the Taiwan Stroke Registry, ischemic stroke is defined as the “acute onset of neurological deficits with signs or symptoms persisting for longer than 24 h, presenting to the hospital within 10 days of onset, presence or absence of acute ischemic lesion(s) on brain computed tomography, or presence of acute ischemic lesion(s) on diffusion weighted magnetic resonance images that correspond to the clinical manifestations.” The accuracy of recording ischemic stroke diagnoses in the NHIRD is high, and the NHIRD appears to be a valid resource for population-based research on ischemic stroke^11^.

The pure dissection of carotid artery in collagen disorders may induced the stroke. However, these patients were young, rarely with hypertension, hyperlipidemia and gout. Therein, these patients rarely entry into our study. Diagnosis and treatment of non-atherosclerotic stroke relies heavily upon history and physical exam as well as CT/MR and catheter angiography. Treatment depends upon the etiology of the ischemia but is in general focused on managing the underlying condition and reducing the risk of future stroke either through antithrombotic therapy or revascularization^12^.

### The plausible mechanism for stroke prevention

Primary prevention of ischemic stroke includes lifestyle modification and diet, treatment of risk factors including hypertension, hyperlipidemia, and DM, antiplatelet therapy for high cardiovascular risk patients, and anticoagulation in atrial fibrillation. The primary prevention of hemorrhagic stroke include the aggressive treatment of hypertension, restriction in alcohol intake, and occlusion of the left atrial appendage in patients with atrial fibrillation and permanent contraindications for oral anticoagulation^13^. The stains seem to play a role for prevention of the ischemic stroke. In contrast, the role of statins for the risk for hemorrhagic stroke is controversial^13^.

### Definition of colchicine cohort

**Figure 1:**
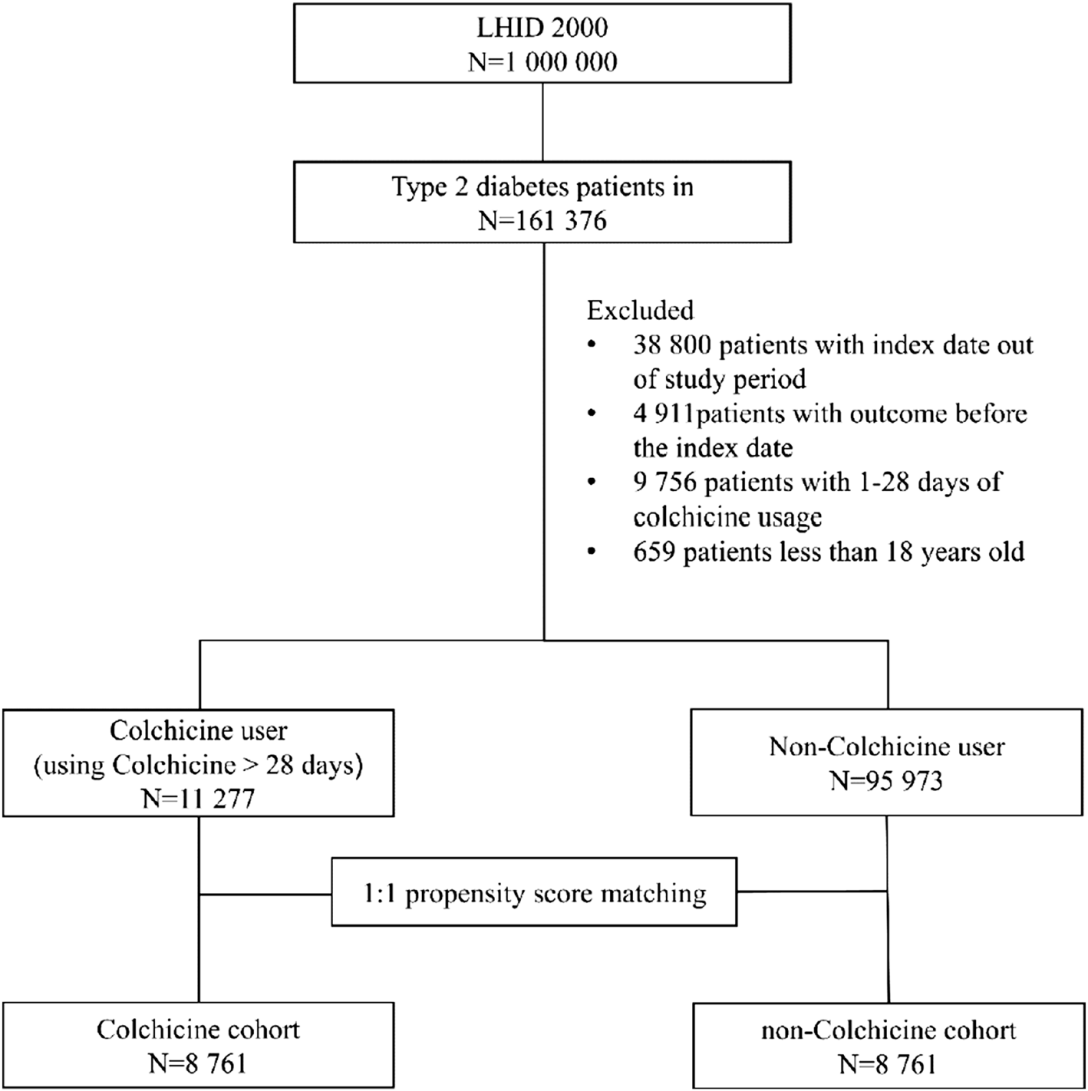
Study flowchart.

From a mechanistic standpoint, the anti‐inflammatory effects of colchicine are not only mediated by direct interaction with microtubules and by regulation in cytokine secretion but also due to modifications at the transcriptional level^14^. Thus, longer therapy durations (such as > 28 days) are necessary to establish the full effects of colchicine; thus, we enrolled patients who had tolerated colchicine for > 28 days^15,16^. In the DM cohort, the colchicine users had higher frequency of pneumonia than nonusers; therefore, we set the 28-day cutoff point to observe tolerance for colchicine^17^. This policy facilitates decision-making for continuation of colchicine use (Fig. 1).

### Propensity score matching

To control for confounding effects, we performed a 1:1 PSM between patients with and without colchicine prescriptions. Logistic regression was used to calculate the propensity score on the basis the following covariates: age, sex, index year, hypertension-related disease (presumed blood pressure, BP ≥ 140/90)^18^, coronary artery disease, acute pericarditis, endocarditis, myocarditis, other disease of pericardium, other disease of endocardium, cardiomyopathy, conduction disorder, cardiac arrhythmia, heart failure, dyslipidemia, hypoglycemia, obesity, gout, liver cirrhosis, hepatitis B, hepatitis C, pneumonia, adapted Diabetes Complications Severity Index (aDCSI score), and inpatient days. The ATC codes used were other antihypertensive agents, diuretics, beta-adrenoceptor blockers, calcium channel blockers, angiotensin-converting-enzyme inhibitors and angiotensin II antagonists, direct renin inhibitors, oral hypoglycemic agents, insulin injection agents, antithrombotic agents (including aspirin, warfarin, heparin, and ticlopidine), NSAIDs, steroids, antigout benzbromarone, allopurinol, and hypolipidemic drugs such as statins, fibrates, bile acid sequestrantse, nicotinic acid and derivates, and other hypocholesterolaemic and hypotrygliceridaemic drugs.

### Diabetes Complications Severity Index score

The aDCSI score was calculated to evaluate the severity of complications among diabetes patients. The severity index included the following complications: nephropathy, retinopathy, peripheral vascular disease, cardiovascular disease, stroke, neuropathy, and metabolic disorders. Renal function was related to colchicine use. Therefore, the components of the aDCSI such as DM nephropathy and renal insufficiency were included in the analysis. Furthermore, aDCSI severity for the variables in the analysis was used to avoid bias due to unequal numbers of male and female enrollees on the risk of stroke^19^.

Because DM-related complications were the major determinant of hospital mortality rather than diabetes per se, glycated hemoglobin (HbA1c) level, or initial blood glucose level, the progression of aDCSI was an accurate predictor of acute coronary syndrome, ischemic stroke, and hemorrhagic stroke. Therefore, aDCSI severity, concurrent medications, and diseases were included in the analyses instead of laboratory tests to evaluate the effects of these clinical factors on stroke incidence^20^. Thus, even without using laboratory data, we could monitor the risk of stroke based on the aDCSI following colchicine use^21^. Wicke et al. reported that higher aDSCI levels are correlated with a higher risk of stroke, supporting our assumption^22^. Moreover, Lo et al. reported that the three variables in the aDSCI—neuropathy, nephropathy, and retinopathy—indicate a higher risk of hemorrhagic stroke (adjusted hazard ratio [aHR] = 4.12) and ischemic stroke (aHR = 2.64)^23^. In our study, patients with hypoglycemia were more likely to have a aDSCI score of 2 (27.10% vs 24.58%) and had higher frequencies of ischemic stroke (11.43% vs 6.75%) and hemorrhagic stroke (2.11% vs 1.84%) among the colchicine users (Appendix Tables 1 and 2). These findings support that the aDSCI is a useful index for predicting the effects of colchicine use on stroke among patients with DM.

### Study outcome

The analyzed outcome in this study was a new diagnosis of stroke during the follow-up period. We classified stroke into two categories: hemorrhagic stroke and ischemic stroke. All patients were followed up from the index date until stroke diagnosis, death, withdrawal from insurance, or the end of 31 December 2013.

### Colchicine prescription

In the colchicine cohort, the history of colchicine use for each patient was measured as the cumulative daily defined dose (cDDD), which was calculated by summing the DDD from the index date to the study endpoint. Dose–response relationships for risk of stroke were evaluated by using the cDDD (none, 14 < cDDD ≤ 20, 21 < cDDD ≤ 50, 51 < cDDD < 150, cDDD > 150), duration of colchicine use (nonusers, 28–60 days, 61–180 days, 181–360 days, > 360 days), and period from last colchicine use to study endpoint (< 30 days, 30–60 days, 61–180 days, > 180 days).

Meanwhile, according to ATC, the cDDD = 1 mg = 0.5MG/tablet × 2. We also calculated cumulative DDD (cDDD) of colchicine per year by summing DDDs prescribed per year per individual, and further classified colchicine users by cDDD per year as follows: (1) > 14 < 20 cDDD (> 28 < 40 tablets) per year, that is, used > 28 < 40 days per year, (2) 21–50 cDDD (41–100 tablets) per year, that is, 41–100 days, (3) 51–150 cDDD (101–300 tablets) per year, that is, 100–300 days, and (4) over 150 cDDD (> 300 tablets) per year, that is, 150 days of using over 1 DDD.

### Colchicine users with gout subcohort

Colchicine users who were with DM and gout both used colchicine and antigout drugs such as “allopurinol-colchicine/nonsteroid anti-inflammatory drug (NSAID),” “benzbromarone–colchicine/NSAID,” “for prevention of gout attacks,” and “NSAID–colchicine” for acute gout attacks or for prevention of chronic gout (Appendix Table 3).

### Colchicine users without gout subcohort

Colchicine users who were with DM with nongout conditions such as arthritis-**c**rystal arthropathies, systemic inflammatory diseases such as sarcoidosis, Behcet's syndrome, autoimmune disease, chronic idiopathic or spontaneous urticarial skin diseases, allergic purpura, psoriasis, collagen vascular diseases, and hypertension-related diseases (Appendix Table 3 and Appendix Table 4)^24–30^.

### Statistical analysis

We compared the demographic characteristics and comorbidities between the colchicine and the comparison cohorts by using the standard mean difference (SMD); values less than 0.1 indicated no significant differences. The incidence of stroke, ischemic stroke, and hemorrhagic stroke was calculated for both cohorts as the number of patients with stroke divided by the sum of person-years (per 1000 person-years). Multivariable Cox proportional hazard models were used to estimate the adjusted hazard ratios (aHR) of stroke after adjustment of potential confounding variables. Ignoring competing risks may lead to an overestimation of the cumulative incidence. Depending on the research question, in the presence of competing events, survival data should be analyzed using either a cause-specific hazard model or a subdistribution hazard model. In this study, we performed the subdistribution hazard model for examining this bias^31^.

We obtained the cumulative incidence curves of stroke, ischemic stroke, and hemorrhagic stroke for the colchicine and the comparison cohorts by using the Kaplan–Meier method and tested the curve differences by using the log-rank test. Further analysis was performed to evaluate the effects of the cDDD, duration of colchicine use, and the last day of colchicine use on the various dose–response categories. All analyses were conducted using SAS statistical software (Version 9.4 for Windows; SAS Institute, Cary, NC, USA). *P* values of < 0.05 indicated statistical significance.

## Results

Table 1 presents the baseline characteristics, comorbidities, and medications of the study patients. After PSM, we identified 8761 colchicine users in the case cohort and 8761 nonusers in the comparison cohort. In the matched cohorts, no significant difference was noted in the distribution of age, sex, comorbidities, aDCSI score, inpatient days, or medications between the colchicine and comparison cohorts. The mean follow-up durations for patients in the colchicine cohort and the comparison cohort were 4.39 ± 3.34 years and 5.24 ± 3.42 years, respectively.

**Table 1:** Baseline characteristics of patients treated with and without colchicine.

Characteristics	Before PS matched				SMD	After PS matched				SMD
	Patients with diabetes					Patients with diabetes				
	Non-colchicine cohort		Colchicine cohort			Non-colchicine cohort		Colchicine cohort		
	n = 95,973		n = 11,277			n = 8761		n = 8761		
	n	%	n	%		n	%	n	%	
**Age, years**										
18–50	25,063	26.11	2436	21.60	0.106	1753	20.01	1873	21.38	0.034
50–65	35,358	36.84	3887	34.47	0.050	3096	35.34	3038	34.68	0.014
> 65	35,552	37.04	4954	43.93	0.141	3912	44.65	3850	43.94	0.014
Mean ± SD	59.44 ± 15.08		61.60 ± 14.18		0.148	62.24 ± 14.31		61.71 ± 14.23		0.037
**Gender**					0.544					0.017
Male	51,372	53.53	3131	27.76		2861	32.66	2793	31.88	
Female	44,601	46.47	8146	72.24		5900	67.34	5968	68.12	
**Comorbidity**										
Hypertension-related disease	62,195	64.80	8796	78.00	0.295	6967	79.52	6878	78.51	0.025
Dyslipidemia	49,954	52.05	6845	60.70	0.175	5436	62.05	5408	61.73	0.007
Hypoglycemia	985	1.03	151	1.34	0.029	122	1.39	107	1.22	0.015
Obesity	2406	2.51	319	2.83	0.020	244	2.79	255	2.91	0.008
Gout	14,433	15.04	7966	70.64	1.358	5751	65.64	5790	66.09	0.009
Liver cirrhosis	3480	3.63	530	4.70	0.054	427	4.87	403	4.60	0.013
Hepatitis B	5660	5.90	478	4.24	0.076	410	4.68	423	4.83	0.007
Hepatitis C	3050	3.18	361	3.20	0.001	309	3.53	291	3.32	0.011
Pneumonia	11,424	11.90	1492	13.23	0.04	1270	14.50	1197	13.66	0.024
**aDCSI**										
0	69,086	71.98	7774	68.94	0.067	5974	68.19	6039	68.93	0.016
1	6566	6.84	706	6.26	0.023	548	6.26	566	6.46	0.008
2	20,321	21.17	2797	24.80	0.086	2239	25.56	2156	24.61	0.022
Inpatient day	9.00 ± 10.25		8.65 ± 9.13		0.102	8.80 ± 9.92		8.54 ± 9.06		0.028
**Drug use**										
Antihypertensive agent	76,131	79.33	9573	84.89	0.146	7677	87.63	7584	86.57	0.032
Hypolipidemic agents	20,313	21.17	3612	32.03	0.248	2809	32.06	2769	31.61	0.010
Oral hypoglycemic agent	22,779	23.73	5560	49.30	0.551	3993	45.58	3895	44.46	0.022
Insulin injection	17,223	17.95	2017	17.89	0.002	1699	19.39	1619	18.48	0.023
Antithrombotic agent	45,031	46.92	6358	56.38	0.190	5161	58.91	5031	57.43	0.030
**Gout drug use**										
Nsaid	94,646	98.62	10,887	96.54	0.135	8696	99.26	8703	99.34	0.010
Steroid	78,889	82.20	9625	85.35	0.086	7562	86.31	7612	86.89	0.017
Allopurinol	7357	7.67	5551	49.22	1.038	3519	40.17	3529	40.28	0.002
Benzbromarone	11,771	12.26	6737	59.74	1.138	4678	53.40	4683	53.45	0.001
Follow-up period, years	4.18 ± 3.15		5.43 ± 3.49		0.377	4.39 ± 3.34		5.24 ± 3.42		0.252

Data shown as n(%) or mean ± SD.

SMD: standard mean difference; PS, propensity score.

Table 2 presents the incidence rates and hazard ratios of stroke, ischemic stroke, and hemorrhagic stroke for the colchicine cohort relative to the comparison cohort. The incidence rates of stroke, ischemic stroke, and hemorrhagic stroke were 16.05, 12.79, and 3.29 per 1000 person-years, respectively, in the colchicine cohort. The corresponding incidence rates in the comparison group were 26.19, 21.33, and 4.86. According to the multivariable Cox proportional hazards models adjusted for age, sex, comorbidities, aDCSI score, inpatient days, and medication, we observed that the risks of stroke (aHR = 0.61, 95% confidence interval [CI] = 0.55–0.67), ischemic stroke (aHR = 0.59, 95% CI = 0.53–0.66), and hemorrhagic stroke (aHR = 0.66, 95% CI = 0.53–0.82) were significantly lower in the colchicine cohort than in the comparison cohort. The subdistribution hazard model revealed the colchicine was associated with lower risk of ischemic stroke and hemorrhagic stroke.

**Table 2:** Incidence and hazard ratios of stroke in DM patients treated with and without Colchicine.

Variables	Before PS matched					After PS matched					aSHR (95% CI)
	N	PY	IR	cHR (95% CI)	aHR (95% CI)	N	PY	IR	cHR (95% CI)	aHR (95% CI)	
**Stroke**											
Colchicine											
No	7816	401,016	19.49	1.00 (reference)	1.00 (reference)	1007	38,452	26.19	1.00 (reference)	1.00 (reference)	1.00 (reference)
Yes	962	61,250	15.71	0.85 (0.79, 0.91)***	0.67 (0.62, 0.72)***	737	45,919	16.05	0.63 (0.57, 0.69)***	0.61 (0.55, 0.67)***	0.70 (0.64, 0.77)***
**Ischemic stroke**											
Colchicine											
No	6442	401,016	16.06	1.00 (reference)	1.00 (reference)	820	38,452	21.33	1.00 (reference)	1.00 (reference)	1.00 (reference)
Yes	774	61,250	12.64	0.83 (0.77, 0.89)***	0.66 (0.6, 0.72)***	586	45,919	12.76	0.61 (0.55, 0.68)***	0.59 (0.53, 0.66)***	0.68 (0.61, 0.76)***
**Hemorrhagic stroke**											
Colchicine											
No	1374	401,016	3.43	1.00 (reference)	1.00 (reference)	187	38,452	4.86	1.00 (reference)	1.00 (reference)	1.00 (reference)
Yes	188	61,250	3.07	0.96 (0.82, 1.12)	0.71 (0.59, 0.84)***	151	45,919	3.29	0.69 (0.55, 0.85)***	0.66 (0.53, 0.82)***	0.77 (0.62, 0.95)*

aHR adjusted for age, sex, hypertension, hyperlipidemia, hypoglycemia, obesity, gout, pneumonia, hepatitis B, hepatitis C, liver cirrhosis, Allopurinol, Benzbromarone, nsaid, Oralsteroid, Antithrombotic, adapted Diabetes Complications Severity Index, aDCSI and inpatient day.

PS, propensity score; N, number of event; PY, person-years; IR, incidence rate, per 1000 person-years; cHR, crude hazard ratio; aHR, adjusted hazard ratio; aSHR: adjusted subdistribution hazard ratio; CI, confidence interval.

*p < 0.05, ***p < 0.001.

We found a significant dose–response effect of colchicine prescription on the reduction of the risk of stroke occurrence. Compared with noncolchicine users, patients who were prescribed colchicine at cDDD = 14–20 (aHR = 0.69, 95% CI = 0.55–0.86), cDDD = 21–50 (aHR = 0.61, 95% CI = 0.55–0.67), cDDD = 51–150 (aHR = 0.62, 95% CI = 0.53–0.71), and cDDD > 150 (aHR = 0.51, 95% CI = 0.44–0.58) had lower stroke risk. Similar results were observed for ischemic stroke (cDDD = 14–20: aHR = 0.70, 95% CI = 0.55–0.90; cDDD = 21–50: aHR = 0.71, 95% CI = 0.60–0.85; cDDD = 51–150: aHR = 0.56; 95% CI = 0.48–0.67; cDDD > 150: aHR = 0.52; 95% CI = 0.45–0.61). Additionally, patients who were prescribed colchicine at the highest cDDD of > 150 had lower hemorrhagic stroke risk (aHR = 0.43, 95% CI = 0.30–0.60) compared with nonusers (Table 3).

**Table 3:** Hazard ratio and 95% CI for stroke associated with total cumulative use of colchicine.

Variables	Before PS matched					After PS matched				
	N	PY	IR	cHR (95% CI)	aHR (95% CI)	N	PY	IR	cHR (95% CI)	aHR (95% CI)
**Stroke**										
Colchicine										
Non-user	7816	401,016	19.49	1.00 (reference)	1.00 (reference)	1007	38,452	26.19	1.00 (reference)	1.00 (reference)
14–20 DDD	94	5127	18.34	0.97 (0.79, 1.19)	0.82 (0.67, 1.01)	79	4515	17.50	0.68 (0.54, 0.85)***	0.69 (0.55, 0.86)**
21–50 DDD	229	11,842	19.34	1.03 (0.90, 1.17)	0.83 (0.72, 0.95)**	190	9921	19.15	0.74 (0.63, 0.86)***	0.74 (0.64, 0.87)***
51–150 DDD	278	18,053	15.40	0.83 (0.73, 0.93)**	0.68 (0.60, 0.77)***	218	13,978	15.60	0.61 (0.52, 0.70)***	0.62 (0.53, 0.71)***
> 150 DDD	361	26,229	13.76	0.75 (0.68, 0.84)***	0.55 (0.49, 0.62)***	250	17,505	14.28	0.56 (0.49, 0.64)***	0.51 (0.44, 0.58)***
p for trend				< 0.0001	< 0.0001				< 0.0001	< 0.0001
**Ischemic stroke**										
Colchicine										
Non-user	6442	401,016	16.06	1.00 (reference)	1.00 (reference)	820	38,452	21.33	1.00 (reference)	1.00 (reference)
14–20 DDD	80	5127	15.60	1.00 (0.81, 1.25)	0.85 (0.68, 1.07)	67	4515	14.84	0.70 (0.55, 0.90)**	0.70 (0.55, 0.90)**
21–50 DDD	178	11,842	15.03	0.97 (0.84, 1.13)	0.78 (0.67, 0.91)**	149	9921	15.02	0.71 (0.60, 0.85)***	0.71 (0.60, 0.85)***
51–150 DDD	214	18,053	11.85	0.77 (0.68, 0.89)***	0.64 (0.56, 0.74)***	161	13,978	11.52	0.55 (0.47, 0.65)***	0.56 (0.48, 0.67)***
> 150 DDD	302	26,229	11.51	0.77 (0.68, 0.86)***	0.57 (0.50, 0.64)***	209	17,505	11.94	0.57 (0.49, 0.67)***	0.52 (0.45, 0.61)***
p for trend				< 0.0001	< 0.0001				< 0.0001	< 0.0001
**Hemorrhagic stroke**										
Colchicine										
Non-user	1374	401,016	3.43	1.00 (reference)	1.00 (reference)	187	38,452	4.86	1.00 (reference)	1.00 (reference)
14–20 DDD	14	5127	2.73	0.84 (0.49, 1.42)	0.68 (0.40, 1.16)	12	4515	2.66	0.55 (0.31, 0.98)*	0.59 (0.33, 1.07)
21–50 DDD	51	11,842	4.31	1.32 (1.00, 1.75)*	1.01 (0.76, 1.36)	41	9921	4.13	0.86 (0.61, 1.20)	0.89 (0.63, 1.24)
51–150 DDD	64	18,053	3.55	1.09 (0.85, 1.40)	0.82 (0.63, 1.08)	57	13,978	4.08	0.84 (0.63, 1.14)	0.84 (0.62, 1.13)
> 150 DDD	59	26,229	2.25	0.72 (0.55, 0.93)*	0.48 (0.36, 0.64)***	41	17,505	2.34	0.50 (0.35, 0.70)***	0.43 (0.30, 0.60)***
p for trend				0.223	< 0.0001				< 0.0001	< 0.0001

aHR adjusted for age, sex, hypertension, hyperlipidemia, hypoglycemia, obesity, gout, pneumonia, hepatitis B, hepatitis C, liver cirrhosis, Allopurinol, Benzbromarone, nsaid, Oralsteroid, Antithrombotic, adapted Diabetes Complications Severity Index, aDCSI and inpatient day.

PS, propensity score; N, number of event; PY, person-years; IR, incidence rate, per 1000 person-years; cHR, crude hazard ratio; aHR, adjusted hazard ratio; CI, confidence interval; DDD: defined daily doses; 14-20DDD: 28–40 tablets, 21-50DD:41–100 tablets, 51-150DDD: 51–300 tablets, > 150DD: > 300 tablets.

*p < 0.01, **p < 0.01, ***p < 0.001.

In the drug duration analysis, the duration of colchicine use was significantly associated with lower risk of stroke (28–60 days: aHR = 0.69, 95% CI = 0.59–0.81; 61–180 days: aHR = 0.66, 95% CI = 0.57–0.76; 181–360 days: aHR = 0.63, 95% CI = 0.52–0.77; > 360 days: aHR = 0.48, 95% CI = 0.41–0.56), ischemic stroke (28–60 days: aHR = 0.67, 95% CI = 0.57–0.80; 61–180 days: aHR = 0.61, 95% CI = 0.52–0.72; 181–360 days: aHR = 0.62, 95% CI = 0.48–0.75; > 360 days: aHR = 0.51, 95%CI = 0.43–0.60), and hemorrhagic stroke (> 360 days: aHR = 0.36, 95%CI = 0.24–0.54) (Table 4).

**Table 4:** Hazard ratio and 95% CI for stroke associated with total duration of colchicine use.

Variables	Before PS matched					After PS matched				
	N	PY	IR	cHR (95% CI)	aHR (95% CI)	N	PY	IR	cHR (95% CI)	aHR (95% CI)
**Stroke**										
Duration of colchicine use										
Non-users	7816	401,016.2	19.49	1.00 (reference)	1.00 (reference)	1007	38,451.89	26.19	1.00 (reference)	1.00 (reference)
28–60 days	237	13,152.66	18.02	0.96 (0.84, 1.09)	0.79 (0.69, 0.90)***	197	11,220.76	17.56	0.68 (0.58, 0.79)***	0.69 (0.59, 0.81)***
61–180 days	300	18,566.81	16.16	0.87 (0.77, 0.97)*	0.72 (0.64, 0.82)***	240	14,637.91	16.40	0.64 (0.55, 0.73)***	0.66 (0.57, 0.76)***
181–360 days	167	9431.19	17.71	0.95 (0.82, 1.11)	0.74 (0.63, 0.87)***	115	6921.28	16.62	0.65 (0.53, 0.78)***	0.63 (0.52, 0.77)***
> 360 days	258	20,099.15	12.84	0.71 (0.62, 0.80)***	0.50 (0.44, 0.57)***	185	13,139.39	14.08	0.55 (0.47, 0.65)***	0.48 (0.41, 0.56)***
p for trend				< 0.001	< 0.001				< 0.001	< 0.001
**Ischemic stroke**										
Duration of colchicine use										
Non-users	6442	401,016.2	16.06	1.00 (reference)	1.00 (reference)	820	38,451.89	21.33	1.00 (reference)	1.00 (reference)
28–60 days	192	13,152.66	14.60	0.95 (0.82, 1.09)	0.78 (0.68, 0.91)**	157	11,220.76	13.99	0.67 (0.56, 0.79)***	0.67 (0.57, 0.80)***
61–180 days	233	18,566.81	12.55	0.82 (0.72, 0.93)**	0.69 (0.60, 0.79)***	183	14,637.91	12.50	0.60 (0.51, 0.70)***	0.61 (0.52, 0.72)***
181–360 days	129	9431.19	13.68	0.90 (0.76, 1.07)	0.71 (0.59, 0.85)***	88	6921.28	12.71	0.61 (0.49, 0.76)***	0.60 (0.48, 0.75)***
> 360 days	220	20,099.15	10.95	0.73 (0.64, 0.83)***	0.52 (0.45, 0.61)***	158	13,139.39	12.02	0.58 (0.49, 0.68)***	0.51 (0.43, 0.60)***
p for trend				< 0.001	< 0.001				< 0.001	< 0.001
**Hemorrhagic stroke**										
Duration of colchicine use										
Non-users	1374	401,016.2	3.43	1.00 (reference)	1.00 (reference)	187	38,451.89	4.86	1.00 (reference)	1.00 (reference)
28–60 days	45	13,152.66	3.42	1.05 (0.78, 1.42)	0.82 (0.61, 1.12)	40	11,220.76	3.56	0.74 (0.52, 1.04)	0.77 (0.55, 1.09)
61–180 days	67	18,566.81	3.61	1.11 (0.87, 1.42)	0.87 (0.67, 1.13)	57	14,637.91	3.89	0.81 (0.60, 1.09)	0.84 (0.62, 1.13)
181–360 days	38	9431.19	4.03	1.26 (0.91, 1.74)	0.89 (0.63, 1.24)	27	6921.28	3.90	0.81 (0.54, 1.22)	0.75 (0.50, 1.13)
> 360 days	38	20,099.15	1.89	0.60 (0.44, 0.83)**	0.39 (0.28, 0.55)***	27	13,139.39	2.05	0.44 (0.29, 0.65)***	0.36 (0.24, 0.54)***
p for trend				0.121	< 0.001				< 0.001	< 0.001

aHR adjusted for age, sex, hypertension, hyperlipidemia, hypoglycemia, obesity, gout, pneumonia, hepatitis B, hepatitis C, liver cirrhosis, Allopurinol, Benzbromarone, nsaid, Oralsteroid, Antithrombotic, adapted Diabetes Complications Severity Index, aDCSI and inpatient day.

PS, propensity score; N, number of event; PY, person-years; IR, incidence rate, per 1000 person-years; cHR, crude hazard ratio; aHR, adjusted hazard ratio; CI, confidence interval.

*p < 0.05, **p < 0.001, ***p < 0.001.

For the period from the last day of colchicine use until the study endpoint, a significantly lower risk of stroke (61–180 days: aHR = 0.63, 95% CI = 0.47–0.84; > 180 days: aHR = 0.30, 95% CI = 0.24–0.39), ischemic stroke (61–180 days: aHR = 0.61, 95% CI = 0.44–0.85; > 180 days: aHR = 0.30, 95% CI = 0.24–0.39), and hemorrhagic stroke (> 180 days: aHR = 0.31, 95% CI = 0.19–0.50) was observed compared with the < 30 days groups (Table 5).

**Table 5:** Hazard ratio and 95% CI for stroke associated with the last day of colchicine use.

Variables	Before PS matched					After PS matched				
	N	PY	IR	cHR (95% CI)	aHR (95% CI)	N	PY	IR	cHR (95% CI)	aHR (95% CI)
**Stroke**										
Last day of colchicine use										
< 30 days	132	3165	41.71	1.00 (reference)	1.00 (reference)	95	2244	42.34	1.00 (reference)	1.00 (reference)
30–60 days	63	1665	37.84	0.91 (0.67, 1.22)	0.88 (0.65, 1.19)	50	1175	42.56	1.00 (0.71, 1.41)	1.04 (0.74, 1.47)
61–180 days	120	4823	24.88	0.59 (0.46, 0.76)***	0.59 (0.46, 0.76)***	90	3363	26.76	0.63 (0.47, 0.83)**	0.63 (0.47, 0.84)**
> 180 days	647	51,597	12.54	0.30 (0.25, 0.36)***	0.30 (0.25, 0.36)***	502	39,137	12.83	0.30 (0.24, 0.37)***	0.30 (0.24, 0.38)***
p for trend				< 0.001	< 0.001				< 0.001	< 0.001
**Ischemic stroke**										
Last day of colchicine use										
< 30 days	110	3165	34.76	1.00 (reference)	1.00 (reference)	76	2244	33.87	1.00 (reference)	1.00 (reference)
30–60 days	51	1665	30.63	0.89 (0.64, 1.24)	0.86 (0.62, 1.21)	41	1175	34.90	1.04 (0.71, 1.51)	1.09 (0.75, 1.60)
61–180 days	97	4823	20.11	0.57 (0.44, 0.75)***	0.57 (0.43, 0.75)***	70	3363	20.81	0.61 (0.44, 0.84)**	0.61 (0.44, 0.85)**
> 180 days	516	51,597	10.00	0.28 (0.23, 0.35)***	0.28 (0.23, 0.35)***	399	39,137	10.19	0.29 (0.23, 0.38)***	0.30 (0.24, 0.39)***
p for trend				< 0.001	< 0.001				< 0.001	< 0.001
**Hemorrhagic stroke**										
Last day of colchicine use										
< 30 days	22	3165	6.95	1.00 (reference)	1.00 (reference)	19	2244	8.47	1.00 (reference)	1.00 (reference)
30–60 days	12	1665	7.21	1.04 (0.51, 2.10)	1.05 (0.52, 2.13)	9	1175	7.66	0.92 (0.42, 2.03)	0.93 (0.42, 2.07)
61–180 days	23	4823	4.77	0.65 (0.36, 1.16)	0.65 (0.36, 1.17)	20	3363	5.95	0.66 (0.35, 1.24)	0.65 (0.34, 1.22)
> 180 days	131	51,597	2.54	0.34 (0.22, 0.54)***	0.36 (0.22, 0.56)***	103	39,137	2.63	0.29 (0.18, 0.48)***	0.31 (0.19, 0.50)***
p for trend				< 0.001	< 0.001				< 0.001	< 0.001

aHR adjusted for age, sex, hypertension, hyperlipidemia, hypoglycemia, obesity, gout, pneumonia, hepatitis B, hepatitis C, liver cirrhosis, Allopurinol, Benzbromarone, nsaid, Oralsteroid, Antithrombotic, adapted Diabetes Complications Severity Index, aDCSI and inpatient day.

PS, propensity score; N, number of event; PY, person-years; IR, incidence rate, per 1000 person-years; cHR, crude hazard ratio; aHR, adjusted hazard ratio; CI, confidence interval.

**p < 0.01, ***p < 0.001.

**Figure 2:**
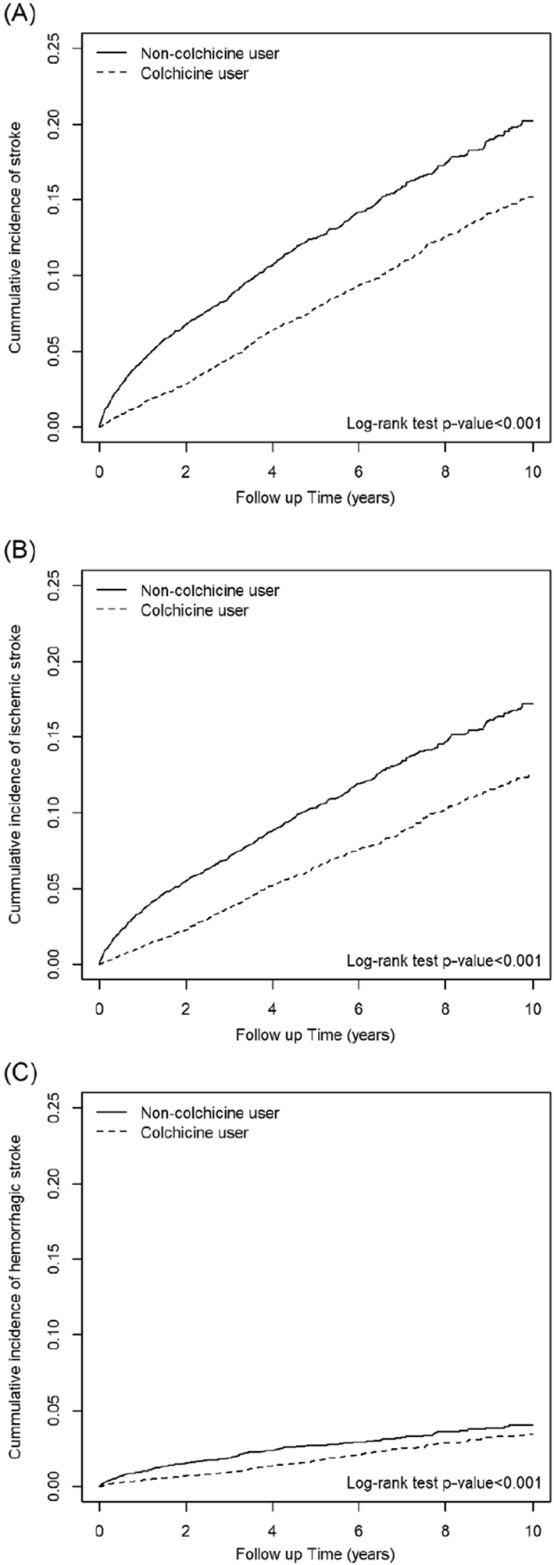
Cumulative incidence of (**a**) stroke, (**b**) ischemic stroke, and (**c**) hemorrhagic stroke between colchicine users and nonusers obtained using the Kaplan–Meier method.

**Figure 3:**
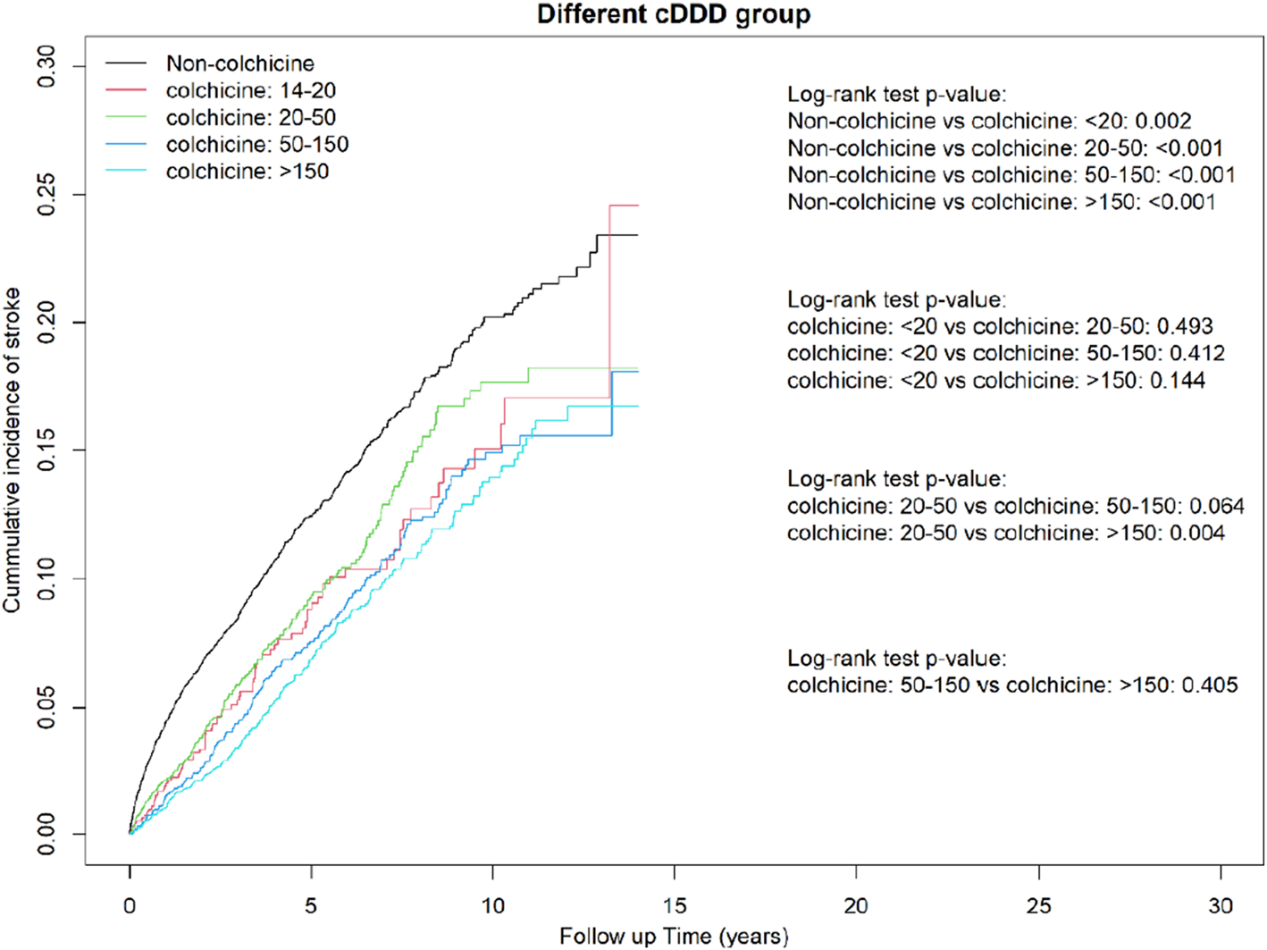
Cumulative incidence of stroke between different cDDD groups of colchicine users and nonusers obtained using the Kaplan–Meier method.

**Figure 4:**
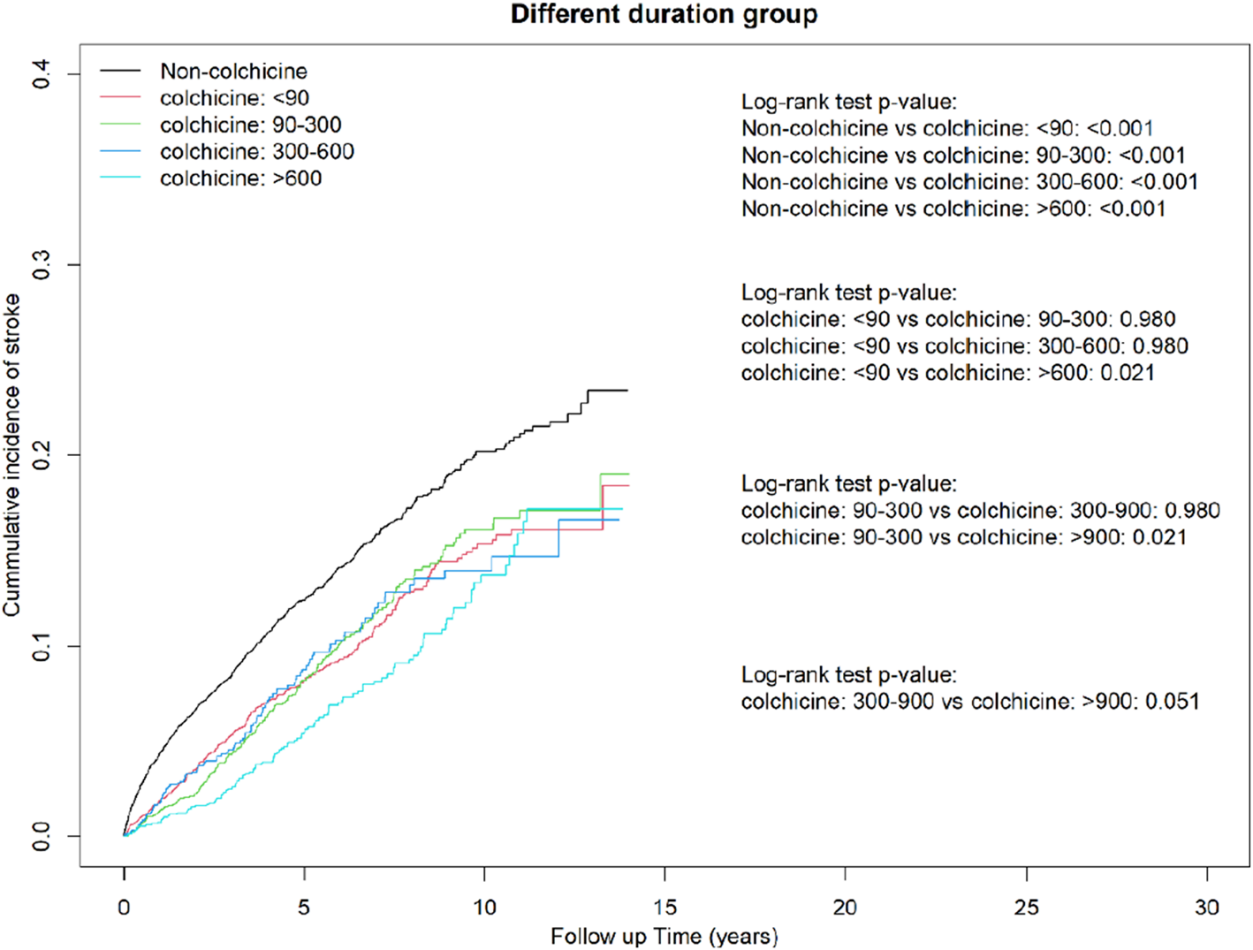
Cumulative incidence of stroke between groups with different colchicine use durations obtained using the Kaplan–Meier method.

In the Kaplan–Meier analysis, the cumulative incidence of stroke, ischemic stroke, and hemorrhagic stroke was significantly lower in the colchicine cohort than in the comparison cohort (log-rank test, *P* < 0.001; Fig. 2A–C). Compared with colchicine nonusers, colchicine users in different cDDD groups and different duration groups had a lower cumulative incidence of stroke during the study period (Figs. 3 and 4).

Table 6 reveals the results of the sensitivity analysis for DM patients without gout. Colchicine use was associated with lower risk of stroke (aHR = 0.64, 95%CI = 0.54–0.75). The aHR of ischemic stroke was 0.64 (95% CI = 0.53–0.76) and that of hemorrhagic stroke was 0.62 (95% CI = 0.42–0.91). The p for interaction of colchicine and gout = 0.116. Subgroup analyses of gout and non-gout did not show an interaction. The subdistribution hazard model revealed the colchicine was associated with lower risk of ischemic stroke. However, colchicine was not associated with the hemorrhagic stroke in DM patients without gout (aHR = 0.69, 95%CI = 0.47–1.00).

**Table 6:** Incidence and hazard ratios of stroke in DM patients without gout treated with and without Colchicine.

Variables	Before PS matched					After PS matched					aSHR (95% CI)
	N	PY	IR	cHR (95% CI)	aHR (95% CI)	N	PY	IR	cHR (95% CI)	aHR (95% CI)	
**Stroke**											
Colchicine											
No	6546	345,069	18.97	1.00 (reference)	1.00 (reference)	345	14,903	23.15	1.00 (reference)	1.00 (reference)	1.00 (reference)
Yes	282	18,362	15.36	0.86 (0.76, 0.97)*	0.69 (0.61, 0.78)***	255	16,208	15.73	0.68 (0.58, 0.80)***	0.64 (0.54, 0.75)***	0.71 (0.60,0.83)***
**Ischemic stroke**											
Colchicine											
No	5402	345,069	15.65	1.00 (reference)	1.00 (reference)	281	14,903	18.86	1.00 (reference)	1.00 (reference)	1.00 (reference)
Yes	236	18,362	12.85	0.87 (0.76, 0.99)*	0.68 (0.6, 0.78)***	209	16,208	12.89	0.69 (0.57, 0.82)***	0.64 (0.53, 0.76)***	0.71 (0.59, 0.85)***
**Hemorrhagic stroke**											
Colchicine											
No	1144	345,069	3.32	1.00 (reference)	1.00 (reference)	64	14,903	4.29	1.00 (reference)	1.00 (reference)	1.00 (reference)
Yes	46	18,362	2.51	0.82 (0.61, 1.10)	0.69 (0.51, 0.94)*	46	16,208	2.84	0.66 (0.45, 0.97)*	0.62 (0.42, 0.91)*	0.69 (0.47,1.00)

aHR adjusted for age, sex, hypertension, hyperlipidemia, hypoglycemia, obesity, gout, pneumonia, hepatitis B, hepatitis C, liver cirrhosis, Allopurinol, Benzbromarone, nsaid, Oralsteroid, Antithrombotic, adapted Diabetes Complications Severity Index, aDCSI and inpatient day.

PS, propensity score; N, number of event; PY, person-years; IR, incidence rate, per 1000 person-years; cHR, crude hazard ratio; aHR, adjusted hazard ratio; aSHR: adjusted subdistribution hazard ratio; CI, confidence interval.

*p < 0.05, ***p < 0.001.

### High frequency of gout in colchicine users with DM

DM and gout had a bidirectional relationship. The frequency of gout in patients with DM before the PSM was 20.88% (7996 + 14,433)/(11,277 + 95,973)^32^. Colchicine is commonly used for gout management in Taiwan. Therefore, the frequency of gout in the colchicine use cohort was high—up to 66.9% after PSM. Tung et al. suggested that gout is a risk factor for DM, and women with gout have a significantly higher risk of DM than men do^33^. Consistent with this assertion, a higher proportion of women (68%) than men (32%) had gout in our cohort. Wijnands et al. also noted that DM comorbidities such as hypertension and obesity were also key risk factors for gout^34^. One explanation is that hypertension, dyslipidemia, low dose aspirin use, and diuretic use may induce hyperuremia, and fluctuations in uric acid may trigger gout attacks. These attacks may be accompanied by sugar fluctuations, leading to stroke^29,32,35–37^. In this study, the high frequency of hypertension-related diseases (78.51%), drug use (86.57%), and dyslipidemia (61.73%) are consistent with the high incidence of gout (66.09%) in the DM cohort^38^. Moreover, the sensitivity analysis indicated a high incidence of hypertension-related diseases in the DM with gout subcohort (75.13%) and the DM without gout subcohort (74.15%), supporting these assertions (Appendix Table 3 and Appendix Appendix Fig. 1).

### Cumulative incidence curves of stroke for the cDDD groups and duration groups and immortal time bias

To account for the immortal time bias, we defined the index date for the case cohort as the first date of colchicine prescription after a diagnosis of DM, and we restricted the case cohort to patients who used colchicine for more than 28 days. Meanwhile, the index date for the control cohort was a randomly assigned date after a diagnosis of DM and was matched to the case patients by propensity score. Moreover, the impact of pay-for-performance (P4P) programs for DM, including the initial enrollment visit, continuing care visit), and annual evaluation visit was analyzed. These strict policies were used to avoid immortal time bias^39^. To confirm the absence of bias, we determined the cumulative incidence curves of stroke for each cDDD group and each duration group. The results revealed that high-dose colchicine users had a lower risk of stroke (Figs. 3 and 4).

### Colchicine use and nephropathy

For the variables, score for chronic renal failure, renal failure not otherwise specified, renal insufficiency, and serum creatinine > 2.0 mg/dL was 2 and that for diabetic nephropathy, acute glomerulonephritis, nephrotic syndrome, hypertension nephrosis, chronic glomerulonephritis, nephritis or nephropathy, urine protein ≥ 30 mg/g of creatinine, or (+) dipstick protein or serum creatinine ≥ 1.5 mg/dL was 1. Most colchicine users (68.93%) and noncolchicine users (68.19%) had a total score of 0 (SMD < 0.1). We thus assumed that most colchicine users had creatinine levels < 1.5 mg/dL (estimated glomerular filtration rate (eGFR) > 50–60 mL/min/1.73 m^2^) and did not have proteinuria. Renal function, as indicated by impairment (eGFR < 60 mL/min/1.73 m^2^ or proteinuria) is associated with stroke risk^40^; because the majority of the cohort did not have these indicators, renal function impairment did not have a significant impact on stroke risk in this study. Long-term use of low doses of colchicine is relatively safe; the dose only requires adjustment if creatinine levels reach 2 mg/dL (eGFR ≈ 30–50 mL/min/1.73 m^2^)^41^. Moreover, P4P for the DM multiple disciplinary team is key to monitoring creatinine level fluctuations in DM patients and facilitates recovery of renal function in patients using colchicine^42^. These strict policies reduce the influence of the confounding factor of renal function impairment on stroke risks in the DM cohort.

## Discussion

The two key findings of this study are as follows: first, the colchicine cohort had a lower risk of stroke, ischemic stroke, and hemorrhagic stroke than the noncolchicine cohort did; patients with the longest durations of colchicine use had the lowest risk of ischemic and hemorrhagic stroke. Moreover, during the follow-up period, significant differences were observed in the cumulative incidence of stroke, ischemic stroke, and hemorrhagic stroke. Second, similar results were observed for colchicine users in the gout and nongout subcohorts. Patients in the nongout subcohort also had a lower risk of stroke, ischemic stroke, and hemorrhagic stroke. Studies have suggested that in populations with a high cardiovascular risk (e.g., patients with DM, gout, hypertension, and coronary artery disease), colchicine use results in a significant reduction of stroke risk, in line with our results^3,5,15,43^ (Appendix Fig. 1).

Pandey et al. reported that higher CRP levels, higher scores on the National Institutes of Health Stroke Scale, high BP, high blood sugar, and higher frequency of hypoglycemia were associated with hemorrhagic stroke^2,37,44^. These higher levels of inflammation markers in patients with DM contribute to the necessity of higher colchicine doses to attenuate severe inflammation and prevent hemorrhagic stroke^37,44^. Furthermore, only the group with cDDD > 150 and duration of use > 360 days had a low risks of hemorrhagic stroke. These findings imply a delayed response in the prevention of hemorrhagic stroke in the DM with colchicine use group^39^. However, the mechanism underlying this effect warrants further research.

Overall, the dose-dependent effect of colchicine use on stroke indicates that colchicine attenuates the risk of stroke, ischemic stroke, and hemorrhagic stroke in patients with complicated DM and high aDCSI scores. Patients with the longest duration of colchicine use had the lowest risk of ischemic stroke and hemorrhagic stroke. A possible explanation for this finding is that these patients have more frequent medical service use and undergo regular follow-up visits in Taiwan^39,45^.

Notably, compared with last day colchicine use < 30 days, the group with last day colchicine use of 30–60 days did not exhibit a significant reduction in risk of stroke or ischemic stroke. For example, patients using colchicine for the management of acute gout cases did not have a reduced risk of stroke^27^. By contrast, patients with last day colchicine use > 180 days still had a lower risk of stroke, ischemic stroke, and hemorrhagic stroke. These results indicate colchicine’s role in the prevention of stroke among patients with DM. Therefore, colchicine may be more effective when administered as a prophylactic such as in long-term therapy (e.g., use days > 360 days and cDDD > 150) than as a treatment agent^3^. Meanwhile, low dose (0.5 mg/d) with low cDDD (> 14 < 20) and low duration use (> 28 < 60 days) with lower risk of ischemic stroke. And as such in this low dose, it is unlikely to increase the risk of intracranial or extracranial bleeding in this vulnerable patient population. If the colchicine is proven to safe and effective, thus this low-cost approach can have a potential to change clinical practice.

Lai et al. that physiological concentrations of serum uric acid displayed anti-inflammatory and chondroprotective effects both in vitro and in vivo^46^. However, Mohsin et al. that high uric acid level may be considered as a risk factor in patients with acute ischemic stroke^47^. Our result reveal that the colchicine use was associated with the lower risk of ischemic stroke regardless with gout or not. These findings should be confirmed in further large-scale randomized controlled trials.

## Strengths

First, this is the first large-scale study to investigate the effects of colchicine use on ischemic and hemorrhagic stroke risk among patients with DM based on their propensity scores for a long duration: 5.24 ± 3.42 years in the colchicine use cohort and 4.39 ± 3.34 years in the noncolchicine use cohort. Second, PSM ensured robust internal validity^48^. Third, comorbidities such as hypertension, hyperlipidemia, gout, and obesity were investigated in this study instead of lifestyle factors; due to the intersection of environmental air pollution, pneumonia, and inflammation, pneumonia was used to represent environmental and economic status; and aDCSI replaced drug adherence based on the closely monitoring the drug compliance of the DM cohort^49^. This approach was applied to avoid interactions between confounding factors. Fourth, aDCSI is a useful tool for predicting the risk of stroke. Variables including the aDCSI score in the NHIRD are similar to those used by studies worldwide, and aDCSI was demonstrated to have external validity^50^. The index includes biochemical data—such as hbA1c levels, cholesterol levels, triglyceride levels, and body mass index (BMI)—which are related to uric acid levels. Thus, aDCSI is suitable for representing data unavailable in the NHIRD, such as uric acid level, smoking status, and alcoholism^49^.

Fifth, according to Dhillon et al., fewer diabetes complications, lower diabetes severity, stricter medication adherence, and psychosocial well-being are direct or indirect predictors of better quality of life among patients with DM^49^. In summary, aDCSI is a tool for the analysis of lifestyle factors—such as smoking, alcohol consumption, exercise, and psychiatric status—for which data are unavailable in the NHIRD. Moreover, aDCSI may be a predictor of hypoglycemia^36^. Thus, we adopted aDCSI in the present study to improve the results; this observation could be used in future studies on the relationship between stroke risk and colchicine use among patients with DM.

Sixth, in the COVID-19 pandemic era, the colchicine use in the patients having virus with diabetes or hypertension is an important topic. Our study may infer to the investigation of the relationship between the stroke and the colchicine use in the diabetes with gout or without gout in future.

## Limitations

This study had several limitations. First, the NHIRD does not provide biochemical data regarding cytokine interleukin or lifestyle data such as smoking status. Second, some patients may not have taken their prescribed medication or may not have taken the prescribed dose, leading to exposure misclassification. This misclassification, if nondifferential, tends to result in hazard ratio underestimation and may explain the lack of associations between stroke risk reduction and colchicine use in some of our findings. The identification of patients with stroke in this study was based on recorded diagnoses or treatment with colchicine rather than a screening of the study population, leading to an underestimation of cases of stroke. Moreover, some patients in the noncolchicine cohort may have had undiagnosed stroke, which would also have resulted in an underestimation of the association with colchicine. Third, although our analysis included a wide range of potential confounding factors, our observational study still had potential residual confounders and indication bias. We aimed to reduce protopathic bias by excluding patients with stroke diagnosis before DM and patients using colchicine. Fourth, the symptoms of hypoglycemia may be similar to those of stroke; however, whether hypoglycemia is a prodromal symptom or a risk factor for stroke remains controversial. Fifth, the HBA1C data and subclassification for chronic kidney disease are unavailable in the NHIRD. Because renal function impairment is a factor for stroke, we replaced the level of renal function impairment with the variables of nephropathy in the aDSCI. A comparison of the frequency of score distributions for scores of 0, 1, and 2 for the variables of nephropathy between the colchicine and noncolchicine groups revealed no significant differences. Altogether, generalization of the results of this study to patients with renal function impairment requires further research.

Sixth, the anti-hypertension drugs have many indications. For example, the beta-blocker is a prescription medicine used to treat migraine, coronary artery disease, cardiac arrhythmia, heart failure and hyperthyroidism. The calcium-blocker was used for the coronary artery disease, pulmonary hypertension. The diuretic was used for edema, heart failure or liver cirrhosis. Thus, the rate of antihypertensive medications (79.3%) was larger than the number of hypertensive-related patients (64.8%). Despite discrepancies in international guidelines, the mantra that every physician should be resumed in “treat the elderly DM patient, not the HbA1c level”. The use of antidiabetic medications (OHA or insulin) in elderly ≥ 75 years may be limited. For example, in elderly DM who have multiple chronic diseases (aDSCI ≥ 1), mild to moderate dementia, and shortened life expectancy, HbA1c should target 7.1–8%, fasting and pre-meal plasma glucose at 90–150 mg/dl, and overnight plasma glucose at 100–180 mg/dl. Thus, if those elderly patients with DM could achieve the target level such as (HBA1c, 6.5–7.5% in healthy older adults, 7.1–7.8% in heart failure, 7.5–8.5% in frail) after lifestyle therapies, the antidiabetic medications may be not necessary in select case such as residential aged care facilities^51–54^. Meanwhile, the frail elderly may be censored or dead before starting use of antidiabetic medications. Moreover, Huang et al. reported about 9.8% of DM patients receiving the tradition Chinese drugs were without antidiabetic medications^55^. Therefore, the high rate of the elderly (44%) and chronic illness (31%) with a higher score of aDSCI (≥ 1, mild to severe frail) in this study may explain the not all DM patients received the antidiabetic medications. In Taiwan, most people take small amounts of colchicine regularly for a long time (> 360 days) to prevent severe attacks or other problems caused by inflammation. For example, people with frequent acute flare-ups or chronic gout tend to use it on a long-term basis. However, the levels of BP, HBA1c, sugar and uric acid were unavailable in NHIRD. These confounding factors were another limitations in this study.

## Conclusion

Colchicine use (cDDD > 14, duration > 28 days) was associated with lower risk of stroke and ischemic stroke in patients with DM. Additionally, cDDD > 150, duration > 360 days was an auxiliary protective factor for stroke, ischemic stroke, and hemorrhagic stroke in these patients. However, the subdistribution hazard model reveal the colchicine was not associated with the hemorrhagic stroke in DM patients without gout.

## Supplementary Information

Supplementary Table 1.

Supplementary Table 2.

Supplementary Table 3.

Supplementary Table 4.

Supplementary Legends.

Supplementary Figure 1.

## Abbreviations

aHR: Adjusted hazard ratio
CI: Confidence interval
DM: Diabetes mellitus
TIA: Transient ischemic attack
LHID2000: Longitudinal Health Insurance Database 2000
NHIRD: National Health Insurance Research Database
ICD-9-CM: International Classification of Diseases, 9th Revision, Clinical Modification
aDCSI: Adapted Diabetes Complications Severity Index
