# Supplementary material for: Effects of colchicine use on ischemic and hemorrhagic stroke risk in diabetic patients with and without gout
Source: Sci Rep. 2022 Jun 2;12:9195. doi: 10.1038/s41598-022-13133-0 (PMC9160857; doi:10.1038/s41598-022-13133-0)
Supplement: Supplementary file 1 — Supplementary Table 1. [file 41598_2022_13133_MOESM1_ESM.docx]

Appendix Table 1. Hypoglycemia were more likely to have a aDSCI score of 2 among the colchicine users.

|  | **Hypoglycemia** | | |  |
| --- | --- | --- | --- | --- |
|  | **no** |  | **yes** |  |
|  | **n (%)** |  | **n (%)** | **p-value** |
| ***All subject*** | N=17293 |  | N=229 | 0.001 |
| **aDCSI score** |  |  |  |  |
| 0 | 11839 (68.46) |  | 174 (75.98) |  |
| 1 | 1112 (6.43) |  | 2 (0.87) |  |
| ≥2 | 4342 (25.11) |  | 53 (23.14) |  |
| ***Among Colchicine user*** | N=8654 |  | N=107 | 0.144 |
| **aDCSI score** |  |  |  |  |
| 0 | 5963 (68.90) |  | 76 (71.03) |  |
| 1 | 564 (6.52) |  | 2 (1.87) |  |
| ≥2 | 2127 (24.58) |  | 29 (27.10) |  |
| aDCSI: adapted Diabetes Complications Severity Index | | | |  |
