# Supplementary material for: Effects of colchicine use on ischemic and hemorrhagic stroke risk in diabetic patients with and without gout
Source: Sci Rep. 2022 Jun 2;12:9195. doi: 10.1038/s41598-022-13133-0 (PMC9160857; doi:10.1038/s41598-022-13133-0)
Supplement: Supplementary file 2 — Supplementary Table 2. [file 41598_2022_13133_MOESM2_ESM.docx]

Appendix Table 2. Patients with hypoglycemia had higher frequencies of ischemic stroke and hemorrhagic stroke among the colchicine users.

|  |  | **Stroke** | **Ischemic** | **Hemorrhagic** |
| --- | --- | --- | --- | --- |
|  | **N** | **n (%)** | **n (%)** | **n (%)** |
| ***Among Colchicine user*** | | |  |  |
| **aDCSI score** |  |  |  |  |
| 0 | 6039 | 492 (8.15) | 386 (6.51) | 106 (1.88) |
| 1 | 566 | 23 (4.06) | 18 (3.21) | 5 (0.91) |
| ≥2 | 2156 | 222 (10.30) | 182 (8.60) | 40 (2.03) |
| p-value |  | <0.001 | <0.001 | 0.222 |
| **Hypoglycemia** |  |  |  |  |
| no | 8505 | 723 (8.35) | 574 (6.75) | 149 (1.84) |
| yes | 105 | 14 (13.08) | 12 (11.43) | 2 (2.11) |
| p-value |  | 0.080 | 0.058 | 0.851 |
| aDCSI: adapted Diabetes Complications Severity Index | | | | |
