# Supplementary material for: Effects of colchicine use on ischemic and hemorrhagic stroke risk in diabetic patients with and without gout
Source: Sci Rep. 2022 Jun 2;12:9195. doi: 10.1038/s41598-022-13133-0 (PMC9160857; doi:10.1038/s41598-022-13133-0)
Supplement: Supplementary file 3 — Supplementary Table 3. [file 41598_2022_13133_MOESM3_ESM.docx]

Appendix Table 3. Colchicine users with gout subcohort.

|  | **Colchicine users** | | |
| --- | --- | --- | --- |
|  | **N=8761** | | |
|  | **Gout** |  | **Non-Gout** |
|  | **N=5790 (66.09%)** |  | **N=2971 (33.91%)** |
| **ICD-9-CM code** | **n (%)** |  | **n (%)** |
| 712 | 338 (5.84) |  | 64 (2.15) |
| 135 136.1 279.49 287.0 696.0 696.1 696.8 708.1 708.8 708.9 710.0 710.1 710.2 710.3 710.9 | 110 (1.90) |  | 66 (2.22) |
| 401-405 | 4350 (75.13) |  | 2203 (74.15) |
| 420-424 | 488 (8.43) |  | 256 (8.62) |
| 425-428 | 1648 (25.46) |  | 810 (27.26) |
| 410-414 | 636 (10.98) |  | 342 (11.51) |
