# Supplementary material for: Effects of colchicine use on ischemic and hemorrhagic stroke risk in diabetic patients with and without gout
Source: Sci Rep. 2022 Jun 2;12:9195. doi: 10.1038/s41598-022-13133-0 (PMC9160857; doi:10.1038/s41598-022-13133-0)
Supplement: Supplementary file 4 — Supplementary Table 4. [file 41598_2022_13133_MOESM4_ESM.docx]

Appendix Table 4. ICD-9-CM code.

| **ICD-9CM** | **Comorbidities** |
| --- | --- |
| 401-405 | hypertension |
| 410–414 | coronary artery disease |
| 420 | acute pericarditis |
| 421 | endocarditis |
| 422 | myocarditis |
| 423 | other disease of pericardium |
| 424 | other disease of endocardium |
| 425 | cardiomyopathy |
| 426 | conduction disorder |
| 427 | cardiac arrhythmia |
| 428 | heart failure |
| 272 | dyslipidemia |
| 251.0, 251.2 | hypoglycemia |
| 278.00 | obesity |
| 274 | gout |
| 571 | liver cirrhosis |
| 070.2, 070.3 | hepatitis B |
| 070.44,070.54 | hepatitis C |
| 486 | pneumonia |
| 712 | **c**rystal arthropathies |
| 135 | sarcoidosis |
| 136.1 | Behcet's syndrome |
| 279.49 | autoimmune disease |
| 708.1, 708.8, 708.9 | chronic idiopathic or spontaneous urticarial skin diseases |
| 287.0 | allergic purpura |
| 696.0, 696.1, 696.8 | psoriasis |
| 710.0, 710.1, 710.2, 710.3, 710.9 | collagen vascular diseases |
| 581.1-585.5 | chronic kidney disease |
| ATC codes | Drug name |
| CO2 | antihypertensive agents |
| C03 | diuretics |
| C07A | beta-adrenoceptor blockers |
| C08C, CO8D, C08E | calcium channel blockers |
| CO9A | angiotensin-converting-enzyme inhibitors |
| C09C | angiotensin II antagonists |
| C09X | direct renin inhibitors |
| A10B | oral hypoglycemic agents |
| A10A | insulin injection agents |
| M01A | NSAIDs |
| B01A | antithrombotic agents (including aspirin, warfarin, heparin, and ticlopidine) |
| D07AC15, R01AD05, H02AB02, D07AC17, S01BA02, H02AB04, H02AB06, H02AB08, and S01BA02 | steroid |
| M04A | antigout benzbromarone, allopurinol |
| C10AA | statins |
| C10AB | fibrates |
| C10AC | bile acid sequestrantse |
| C10AD | nicotinic acid and derivates |
| C10AX | other hypocholesterolaemic and hypotrygliceridaemic drugs |
| Package |  |
| P1401C | initial enrollment visit |
| P1402C | continuing care visit |
| P1403C | annual evaluation visit |
