# Supplementary material for: Effects of colchicine use on ischemic and hemorrhagic stroke risk in diabetic patients with and without gout
Source: Sci Rep. 2022 Jun 2;12:9195. doi: 10.1038/s41598-022-13133-0 (PMC9160857; doi:10.1038/s41598-022-13133-0)
Supplement: Supplementary file 5 — Supplementary Legends. [file 41598_2022_13133_MOESM5_ESM.docx]

**Appendix Legends:**

Appendix Figure. Patients in the nongout subcohort also had a lower risk of stroke, ischemic stroke, and hemorrhagic stroke.
