# Supplementary figures and images for: Effects of colchicine use on ischemic and hemorrhagic stroke risk in diabetic patients with and without gout
Source: Sci Rep. 2022 Jun 2;12:9195. doi: 10.1038/s41598-022-13133-0 (PMC9160857; doi:10.1038/s41598-022-13133-0)

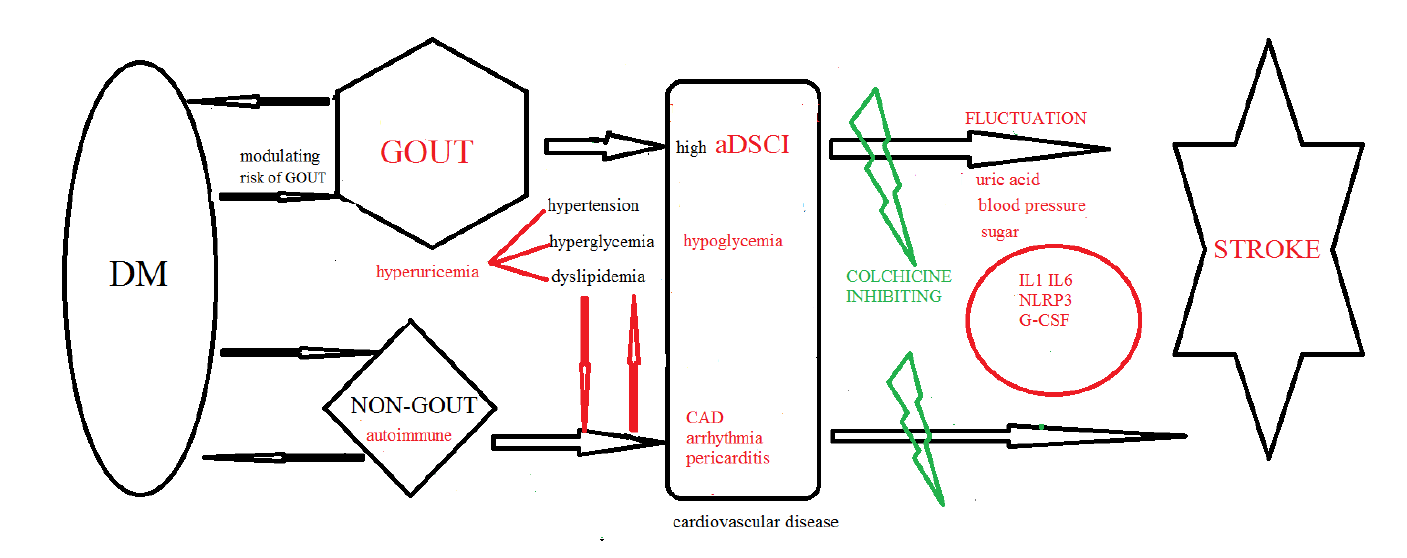

Supplement: Supplementary file 6 — Supplementary Figure 1. [file 41598_2022_13133_MOESM6_ESM.tif]
